# Overexpression of MMP-9 and Its Inhibitors in Blood Mononuclear Cells after Myocardial Infarction - Is It Associated with Depressive Symptomatology?

**DOI:** 10.1371/journal.pone.0105572

**Published:** 2014-08-25

**Authors:** Simon Jönsson, Anna K. Lundberg, Lena Jonasson

**Affiliations:** Department of Medical and Health Sciences, Division of Cardiovascular Medicine, Linköping University, Linköping, Sweden; University of Patras, Greece

## Abstract

**Background:**

Matrix metalloproteinase (MMP)-9 may play a central role in the development and progression of atherosclerosis. Emerging evidence also indicates an association between MMP-9 and depressive symptomatology. Here, we investigated whether expression of MMP-9 and its inhibitors in blood mononuclear cells and plasma were related to depressive symptoms in patients with a recent myocardial infarction (MI).

**Methods and Results:**

Blood sampling was performed between 6 and 18 months after MI in 57 patients. Forty-one clinically healthy subjects were included as controls. Gene expression of MMP-9 and its main tissue inhibitors TIMP-1 and -2 were analyzed in freshly isolated or cultured blood mononuclear cells. Corresponding protein levels were assessed in cell supernatants and plasma. In post-MI patients, mRNA levels of MMP-9 and TIMP-1 and -2 were significantly higher than in controls while protein levels in cell supernatants and plasma did not differ between groups. The Center for Epidemiological Studies - Depression (CES-D) scale was used to assess depressive symptomatology. Repeated assessments during the first 18 months after MI showed significantly higher CES-D scores in patients compared with controls. However, there were no relationships between depressive mood and any of the measurements of MMP-9 or TIMPs.

**Conclusion:**

Our findings indicate that overexpression of MMP-9 and TIMPs in blood mononuclear cells and elevated depressive symptoms represent two unrelated phenomena after MI.

## Introduction

Matrix metalloproteinases (MMPs) are a family of zinc-dependent enzymes involved in extracellular matrix remodeling and leukocyte recruitment to inflammatory sites, thus acting as important modulators of inflammation. MMPs are strictly regulated by endogenous tissue inhibitors of metalloproteinases (TIMPs), and excessive or imbalanced MMP secretion has been related to tissue damage in several inflammatory disorders. Atherosclerosis shares several characterstics with a chronic inflammatory process and MMP-9 is considered to play a key role in disease progression and vulnerability to plaque rupture [1], [2]. Overexpression of MMP-9 is not only detected in atherosclerotic lesions but also in peripheral blood. Patients with acute myocardial infarction (MI) exhibit increased expression of MMP-9 mRNA in blood monocytes. Furthermore, increased expression of MMP-9 has been detected in blood mononuclear cells of patients with stable CAD and patients with advanced carotid disease. A number of studies have also shown that plasma levels of MMP-9 in CAD patients are associated with plaque progression and recurrent cardiac events [3], [4].

Lately, MMP-9 has emerged as a candidate marker of depression. A significant association between depressive symptoms and MMP-9 levels in plasma was recently demonstrated in a Swedish middle-aged normal population [5]. Moreover, MMP-9 in plasma was highlighted as one of the strongest markers of major depression when a large protein profiling investigation was performed in order to identify novel biomarkers of psychiatric disorders [6]. Interestingly, Lutgendorf et al. [7] reported that ovarian cancer patients with elevated depressive symptoms had increased levels of MMP-9 in tumor-associated macrophages compared with non-depressed patients.

The prevalence of depression and elevated depressive symptoms is higher in patients with a manifest coronary artery disease (CAD) than in the general population and depressive symptoms in CAD patients are also associated with increased risk of recurrent cardiac events [8]. The underlying mechanisms are still not clarified but it is widely discussed that inflammation is a potential link between depression and CAD. A number of studies in various populations have reported correlations between depression and inflammatory markers, such as C-reactive protein and interleukin(IL)-6, thus giving rise to the “inflammatory hypothesis of depression” [9], [10].

In the present study, we measured the levels of MMP-9 and its main inhibitors TIMP-1 and -2 in blood mononuclear cells and plasma from post-MI patients and healthy controls. Given that MMP-9 may be implicated in the pathogenesis of both depressive and cardiovascular disorders, we also investigated whether there was a relationship between MMP-9/TIMP levels and depressive symptomatology.

## Methods

### Study population

Patients with a recent MI, defined as non-ST-elevation myocardial infarction, according to universally accepted definitions [11], were consecutively recruited from the Outpatients' Cardiology Clinic at the University Hospital in Linköping, Sweden, 4 weeks after the index event. Exclusion criteria were age > 75 years, severe heart failure, neoplastic disease, major clinical depression, chronic immunologic disorders or treatment with immunosuppressive/anti-inflammatory agents. In parallel with recruitment of patients, control subjects from the region were randomly invited from the Swedish Population Register. Subjects who accepted the invitation were included as controls if they were anamnestically healthy. Use of statins or antihypertensive drugs for primary prevention was allowed.

The Center for Epidemiological Studies - Depression scale (CES-D) was used to assess depressive symptomatology [12]. It was designed to measure depressive symptoms in a normal population and is constituted by 20 items with 4 answer categories giving a range of scores from 0 to 60. The Cook-Medley Hostility Scale was used to assess cynical hostility [13]. It is constituted by 12 items with 5 answer categories giving a range of scores from 12 to 60. In patients, CES-D and cynicism were measured on 3 separate occasions; 4 weeks, 12 months and 18 months post-MI. At 4 weeks and 12 months, patients also filled in a questionnaire of mastery (coping) constructed by Pearlin and Schooler [14]. This instrument is constituted by 7 items with 4 answer categories giving a range in the sum score from 7 to 28. In controls, measurements of CES-D and cynicism were performed at the Outpatients' Cardiology Clinic on one occasion.

In patients, blood sampling was performed between 6 and 18 months after the index event when they were in a metabolically stable condition. The study was conducted in accordance with the ethical guidelines of Declaration of Helsinki, and the research protocols were approved by the Ethical Review Board of Linköping University. Written informed consent was obtained from all patients and controls.

### Cell culture

Peripheral blood mononuclear cells (PBMCs) were isolated by density centrifugation (Ficoll-Paque) 400 g×g for 40 min at room temperature (RT). PBMCs were then washed twice in phosphate-buffered saline (PBS) with 0.5% fetal calf serum (FCS) and resuspended in RPMI 1640 medium supplemented with L-glutamine (Gibco by Invitrogen, Carlsbad, CA, USA), 10% FCS, 100 U/ml penicillin and 100 µg/ml streptomycin (Gibco by Invitrogen) to a concentration of 10^6^ cells/ml. The cells were incubated with or without *E. coli* lipopolysaccharide (LPS) (Sigma-Aldrich, St Louis, MO, USA), 100 ng/ml, for 19 h at 37°C in a humidified atmosphere with 5% of CO_2_. Cell supernatants and cells (snap frozen in liquid nitrogen) were collected and stored at −80°C.

### Quantitative Real Time rt-PCR

Total RNA was isolated with MagMAX-96 Total RNA Isolation Kit (Life Technologies, Carlsbad, California, USA) from both freshly isolated PBMCs and cultured PBMCs, according to manufacturer's instructions. RNA (66 ng) was reversed transcribed by using high capacity cDNA reverse transcription kit with an RNAse inhibitor (Life Technologies) according to manufacturer's instructions. cDNA (1 µL) was amplified by RT-PCR reactions with 1× TaqMan Fast Universal PCR Mastermix (Life Technologies) in 96-well plates on an ABI 7500 Sequence Detector with SDS 1.3.1 software. The following TaqMan Gene Expression Assay kits (Life Technologies) were used: MMP-9, Hs00957562_m1; TIMP-1, Hs00171558_m1; TIMP-2, Hs00234278_m1. Eukaryotic 18S rRNA (Part number: 4352930E) with an amplicon length of 187 bp served as endogenous control. The amount of expressed gene was calculated relative to the amount of rRNA in each sample. Standards were used to create a standard curve in each run according to the standard curve method in user bulletin no 2 (Life Technologies). Each sample was run in duplicates and a maximum deviation of 15% was allowed.

### Luminex and ELISA

MMP-9 and tissue inhibitors of metalloproteinase (TIMP)-1 and -2 were analyzed in PBMC supernatants and plasma samples by Luminex Performance Assay Human MMP-9 and Human TIMP Luminex Performance Assay respectively (R&D Systems, Minneapolis MN, USA) according to the protocol provided by the manufacturer. IL-6 in plasma was determined using Quantikine High sensitivity ELISA kit (R&D Systems) according to manufacturer's instructions and the plates were read in a luminometer (Biotek, Germany).The interassay CV were 13% for MMP-9, 6.5% for TIMP1, 14% for TIMP-2 and 7% for IL-6.

### Intracellular assessment of MMP-9 and TIMP-1 by blood flow cytometry

Cells were stained with the following monoclonal antibodies; CD3- APC/H7 clone SK7 (BD Biosciences, San José, CA, US) and CD14-PeCy7 clone M5E2 (Nordic Biosite, Täby, Sweden). Whole blood and antibodies were incubated for 15 minutes at RT, thereafter erythrocytes were lysed with FACS Lysing Solution (BD Biosciences) for 15 minutes at room temperature (RT). The cells were permeabilized for 30 min at RT with Permeabilizing Solution 2 (BD Biosciences) and washed with Permeabilization buffer (Ebioscience, San Diego, CA, USA). Unspecific binding was blocked with 10% FCS and thereafter, cells were stained with antibodies against MMP-9 and TIMP-1, conjugated with FITC and PE respectively, (RnD Systems) for 30 min in 4°C. After washing in Permeabilization buffer followed by resuspension in PBS with 0.5% FCS, cells were analyzed using Beckman Coulter Gallios (Beckman Coulter, Miami Lakes, Florida, US). The obtained data was analyzed and visualized using the Kaluza 1.2 software (Beckman Coulter). When this analysis was performed, antibodies against TIMP-2 were not commercially available.

### Statistics

SPSS 21 was used for statistical analyses. For clinical and laboratory characteristics, data are presented as median (inter-quartile range). The statistical significance of any difference between two groups was tested by using Mann-Whitney U-test. Chi-square test was used for nominal data. Friedman's test was used to compare more than 2 observations repeated on the same subjects and Wilcoxon signed-ranks test was used for pair-wise comparisons. A p-value<0.05 was considered statistically significant while a p-value between 0.05 and 0.1 was considered a trend. When Spearman's rank correlation coefficient was used for correlation analyses, p-value<0.01 was considered significant.

## Results

### Study population

Clinical and biological characteristics of patients and controls are presented in Table 1. There were no differences in age, sex distribution, body mass index, smoking status, plasma IL-6 or leukocyte cell subsets between the groups while parameters such as statin use and lipid profile differed significantly. Table 1 also shows the results of psychological measurements in addition to the number of patients completing each questionnaire on different test occasions. CES-D scores did not vary significantly between different test occasions, neither did cynical hostility or mastery scores. Therefore, arithmetic means of samples from 2 or 3 occasions were used in all statistical analyses. Post-MI patients had significantly higher CES-D scores compared with controls, while cynical hostility did not differ between groups.

**Table 1:** Characteristics of controls and post-MI patients.

	Controls (n = 41)	Post-MI patients (n = 57)	P
***Clinical measurements***			
**Age, years**	67 (66-72)	66 (61-72)	0.16
**Sex (male/female)**	30/11	46/11	0.46
**Body mass index, kg/m^2^**	26 (25-28)	27 (25-30)	0.11
**Smoking, n (%)**	1 (2.4)	6 (12)	0.23
**Diabetes, n (%)**	0 (0)	12 (21)	0.001
**Statin treatment, n (%)**	4 (9.8)	56 (98)	<0.001
**Anti-hypertensive treatment, n (%)**	5 (13)	29 (51)	<0.001
**Coronary angiography, 1/2/3-vessel disease, n**	-	20/19/18	
***Laboratory measurements***			
**Total cholesterol (mmol/l)**	5.4 (4.6-5.9)	3.9 (3.3-4.3)	<0.001
**LDL cholesterol (mmol/l)**	3.1 (2.5-3.9)	2 (1.7-2.5)	<0.001
**HDL cholesterol (mmol/l)**	1.6 (1.4-1.8)	1.1 (1.1-1.4)	<0.001
**Triglycerides (mmol/l)**	1.1 (0.8-1.7)	1.2 (0.9-1.8)	0.38
**IL-6, pg/ml**	2.5 (1.3-3.1)	2.2 (1.6-3.1)	0.97
**Monocytes (×10^6^** **cells/ml)**	470 (380-570)	480 (370-590)	0.66
**T cells (×10^6^** **cells/ml)**	1457 (1202-1624)	1350 (975-1777)	0.18
**Granulocytes (×10^9^** **cells/ml)**	3190 (2154-4659)	3648 (2822-4300)	0.28
***Psychological measurements***			
**CES-D, 4 weeks (n = 57)**	-	7 (3-14)	
**CES-D, 12 months (n = 43)**	-	6 (2-10)	
**CES-D, 18 months (n = 51)**	-	7 (3-14)	
**CES-D^a^**	5 (1-8)	8 (3-13)	0.020
**Cynicism, 4 weeks (n = 54)**	-	28 (21-33)	
**Cynicism, 12 months (n = 44)**	-	28 (24-37)	
**Cynicism, 18 months (n = 51)**	-	27 (21-36)	
**Cynicism^a^**	30 (24-34)	28 (23-34)	0.83
**Coping, 4 weeks (n = 53)**	-	23 (21-26)	
**Coping, 12 months (n = 42)**	-	24 (21-26)	
**Coping** *	-	24 (21-26)	

CES-D, The Center for Epidemiological Studies - Depression scale.

*scores in patients represent mean values derived from 2 or 3 occasions.

Values are given as median (inter-quartile range) or n (%).

### Expression of MMP-9 and endogenous inhibitors in PBMCs

**Figure 1:**
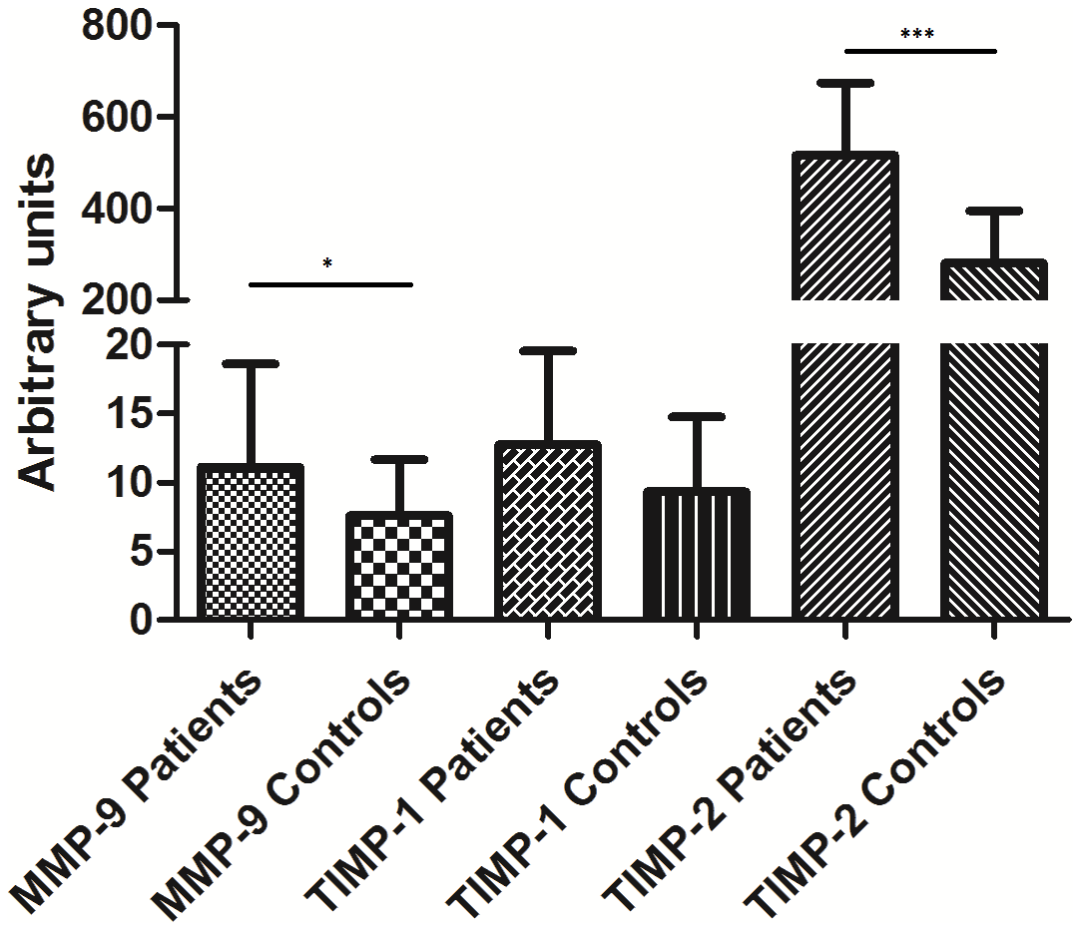
mRNA expression of MMP-9, TIMP-1 and TIMP-2 in freshly isolated PBMCs. Values are given as median (inter-quartile range).

The mRNA levels of MMP-9 and its main endogenous inhibitors TIMP-1 and -2 were analyzed in freshly isolated PBMCs and in PBMCs cultured with or without LPS. In freshly isolated PBMCs, the mRNA expression of MMP-9 was significantly higher in patients than in controls (Figure 1). After 19 h of culture in medium, MMP-9 mRNA levels were markedly increased in both patients and controls although this increase was blunted when LPS was added (Table 2). Compared with controls, patients showed significantly higher levels of TIMP-2 mRNA in freshly isolated PBMCs while TIMP-1 mRNA levels were significantly higher in patients' cells after 19 h of culture. Upon LPS stimulation, TIMP-1 mRNA levels in PBMCs increased while TIMP-2 mRNA levels decreased without any differences between patients and controls. The ratios MMP-9/TIMP-1 and MMP-9/TIMP-2 in freshly isolated PBMCs did not differ between patients and controls.

**Table 2:** mRNA expression and protein secretion of MMP-9, TIMP-1 and -2 in PBMCs cultured for 19 h with or without LPS.

Analyte		PBMCs	Controls (n = 31)	Post MI-patients (n = 55)	P
**MMP-9**	mRNA	19-h culture, medium only	249 (90-350)	298 (149-516)	0.17
		19-h culture, LPS	147 (100-209)*	143 (96-248)***	0.98
	Protein	19-h culture, medium only	8 (4.6-11)	8.3 (5.3-14)	0.77
		19-h culture, LPS	4.8 (3-8.4)*	4.6 (3-6.5)***	0.38
**TIMP-1**	mRNA	19-h culture, medium only	8.7 (6.3-12)	14 (10-22)	<0.001
		19-h culture, LPS	14 (7.8-22)*	17 (10-24)	0.46
	Protein	19-h culture, medium only	14 (5.9-22)	12 (8.7-18)	0.96
		19-h culture, LPS	22 (15-33)***	23 (17-33)***	0.83
**TIMP-2**	mRNA	19-h culture, medium only	429 (347-634)	491 (344-760)	0.35
		19-h culture, LPS	290 (175-392)***	197 (134-324)***	0.069
	Protein	19-h culture, medium only	2.8 (1.5-3.3)	2.2 (1.7-2.6)	0.29
		19-h culture, LPS	2.5 (1.7-3.2)	2.3 (1.7-3.2)	0.48

Values, expressed as arbitrary units for mRNA expression and ng/ml for protein secretion, are given as median (inter-quartile range).

MMP-9, matrix metalloproteinase-9; TIMP-1 and -2, tissue inhibitor of matrix metalloproteinase-1 and -2.

* =  P<0.05, vs 19-h culture, medium only.

***  =  P<0.001, vs 19-h culture, medium only.

### Secretion of MMP-9 and endogenous inhibitors by PBMCs

The secretion of MMP-9 and TIMP-1 and -2 was analyzed in supernatants of PBMCs cultured with or without LPS (Table 2). The secretion of MMP-9 was significantly reduced by LPS. On the other hand, the secretion of TIMP-1 was significantly enhanced by LPS while TIMP-2 levels remained unaffected. Neither the secretion of MMP-9, nor the secretion of TIMP-1 or -2, differed between post-MI patients and controls.

### Plasma levels of MMP-9 and endogenous inhibitors

MMP-9 and TIMP-1 and -2 were also analyzed in plasma samples. The level of MMP-9 did not differ in post-MI patients compared with controls, 50 (38–67) vs 49 (41–81)ng/ml, p = .512, neither did the levels of TIMP-1 and -2, 89 (72–97) vs 92 (80–106)ng/ml, and 84 (71–92) vs 86 (78–92)ng/ml, respectively. The ratios MMP-9/TIMP-1 and MMP-9/TIMP-2 in plasma did not differ between patients and controls.

### Intracellular staining of MMP-9 and TIMP-1

**Figure 2:**
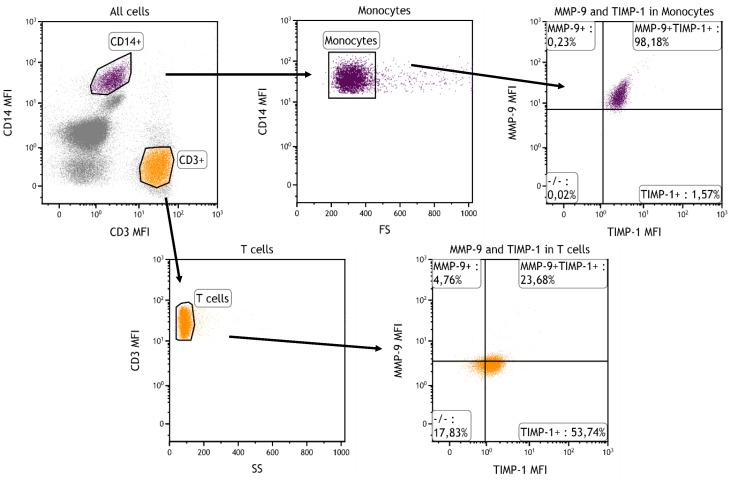
Gating strategy and representative flow cytometry plot of MMP-9-positive and TIMP-1-positive monocytes and T cells.

The presence of MMP-9 and TIMP-1 was assessed in circulating monocytes and T cells by whole blood flow cytometry, see Figure 2. The vast majority of monocytes were MMP-9-positive, 98 (86–100) %, while 19 (3.8–50) % of the T cells showed positive staining for MMP-9. More than 99 % of the monocytes and 84 (79–88) % of the T cells were TIMP-1-positive. In order to perform a semi-quantitative assessment of MMP-9 and TIMP-1 protein, median fluorescence intensities (MFI) of MMP-9 and TIMP-1 were determined related to their own isotype controls. The MFI values of MMP-9 were significantly higher in monocytes than in T cells, 2.8 (2.2–4.6) *vs* 1.4 (1.1–1.6), p<0.001, as were MFI values of TIMP-1, 4.7 (4.4–5.5) *vs* 3.1 (2.9–3.4), p<0.001. There were no differences in MFI between controls and CAD patients.

### Bivariate correlations

In a bivariate correlation analyses including all subjects, the mRNA levels of MMP-9, TIMP-1 and -2 were strongly intercorrelated as were the levels of MMP-9, TIMP-1 and -2 in cell supernatants. In plasma, TIMP-1 and -2, but not MMP-9, were intercorrelated. Circulating levels of MMP-9 and TIMPs did not show any correlations, neither with mRNA levels in PBMCs, nor with amounts released from PBMCs (Table 3). In the whole study population, depressive symptoms also correlated positively with cynical hostility (*r* = 0.267, p<0.01). In the patient population, depressive symptoms correlated negatively with mastery (*r* = −0.594, p<0.001). There were no significant associations between depressive symptoms and measurements of MMP-9, TIMP-1 or -2 at any level (mRNA in PBMCs, protein release from PBMCs or plasma concentrations), neither were there any significant correlations between cynicism or mastery and measurements of MMP-9 or TIMPs. MMP-9 or TIMPs did not correlate with IL-6 in plasma, neither did psychological factors correlate with IL-6 levels.

**Table 3:** Correlation analysis of MMP-9, TIMP-1 and -2 in plasma, mRNA expression in freshly isolated PBMCs and protein secretion from PBMCs incubated for 19 h in medium only.

	Plasma			mRNA			Supernatant		
	MMP9	TIMP1	TIMP2	MMP9 F.	TIMP1 F.	TIMP2 F.	MMP9	TIMP1	TIMP2
**MMP-9 Plasma**									
**TIMP-1 Plasma**	.23								
**TIMP-2 Plasma**	.10	**.69** ***							
**MMP-9 mRNA F.**	.51	-.06	-.04						
**TIMP-1 mRNA F.**	.10	.11	-.01	**.44** ***					
**TIMP-2 mRNA F.**	.10	.10	.05	**.46** ***	**.81** ***				
**MMP -9 Supernatant**	.04	-.18	-.20	-.05	-.08	-.12			
**TIMP-1 Supernatant**	.01	-.12	-.26	-.09	.04	.04	**.51** ***		
**TIMP-2 Supernatant**	-.09	-.11	-.21	-.09	-.01	.05	**.35** ***	**.83** ***	

Spearman's correlation coefficients are presented.

MMP-9, matrix metalloproteinase-9; TIMP-1 and -2, tissue inhibitor of matrix metalloproteinase-1 and -2.

*** p<0.001, F  =  Freshly isolated PBMC.

### Comparison between patients with sustained low or high levels of depressive symptoms

Patients were divided into two groups depending on sustained low or high CES-D scores; those with CES-D≤6 on at least two occasions (n = 24) and those with CES-D≥7 on at least two occasions (n = 27) based on median levels derived from the different test occasions. Patients with sustained elevated depressive symptoms had significantly higher scores of cynical hostility and lower scores of mastery than patients with sustained low CES-D scores. As shown in Table 4, there were no significant differences in clinical characteristics between the 2 groups. Plasma levels of MMP-9 and TIMPs or mRNA levels of MMP-9 and TIMPs in freshly isolated PBMCs did not differ, neither did mRNA levels in cultured PBMCs or release of MMP-9 and TIMPs ex vivo differ between the groups (data not shown).

**Table 4:** Characteristics of post-MI patients with sustained low CES-D scores (≤ 6 on at least 2 occasions) or high CES-D scores (≥ 7 on at least 2 occasions).

	Sustained low CES-D scores (n = 24)	Sustained high CES-D scores (n = 27)	P
***Psychological measurements***			
**CES-D^a^**	3.2 (1.6-4.3)	13 (10-19)	<0.001
**Cynical hostility** *	25 (21-31)	32 (26-36)	0.004
**Coping** *	25 (23-26)	20 (18-26)	<0.001
***Clinical measurements***			
**Age, years**	67 (64-71)	65 (56-69)	0.22
**Sex (male/female)**	18/6	21/5	0.72
**Body mass index, kg/m^2^**	27 (24-30)	28 (25-31)	0.33
**Smoking, n (%)**	2 (8)	3 (12)	0.67
**Diabetes, n (%)**	4 (17)	5 (19)	0.73
**Anti-hypertensive treatment, n (%)**	11 (46)	12 (46)	>0.99
**Coronary angiography, 1/2/3-vessel disease, n**	10/7/7	8/10/9	0.37
***Laboratory measurements***			
**MMP-9, plasma ng/ml**	41 (30-75)	52 (44-67)	0.20
**TIMP-1, plasma ng/ml**	93 (78-100)	84 (70-95)	0.29
**TIMP-2, plasma ng/ml**	86 (77-91)	84 (67-91)	0.27
**MMP-9, mRNA** †	290 (150-520)	220 (120-610)	0.98
**TIMP-1, mRNA** †	16 (12-25)	12 (8-18)	0.11
**TIMP-2, mRNA** †	530 (330-880)	450 (390-560)	0.36
**IL-6, plasma pg/ml**	1.9 (1.4-3.3)	2.1 (1.6-2.8)	0.82

CES-D, The Center for Epidemiological Studies - Depression scale; MMP-9, matrix metalloproteinase-9; TIMP-1 and -2, tissue inhibitor of matrix metalloproteinase-1 and -2.

*  =  scores in patients represent mean values derived from 2 or 3 occasions.

† *=  mRNA values in freshly isolated PBMCs, expressed as arbitrary units.*

Values are given as median (inter-quartile range) or n (%).

## Discussion

The overexpression of MMP-9 mRNA in freshly isolated PBMCs from post-MI patients highlights the presence of ongoing low-grade inflammation in clinically stable conditions of CAD. It supports previous results showing enhanced MMP-9 mRNA expression in PBMCs or isolated monocytes from patients with CAD or carotid atherosclerosis [15]–[17]. This characteristic seems to be shared by other chronic inflammatory disorders since increased expression of MMP-9 mRNA in blood mononuclear cells has been demonstrated in multiple sclerosis [18] and systemic lupus erythematosus [19]. Likewise, our findings that post-MI patients exhibited increased TIMP-1 and -2 mRNA levels in PBMCs are in line with previous studies of patients with multiple sclerosis [18], [20]. TIMP-1 and -2 belong to the family of endogenous inhibitors that regulates MMP-9 activity. Although TIMP-1 is often mentioned as the main inhibitor, TIMP-2 has been shown to be more effective than TIMP-1 in inhibiting MMP-9 [21], [22]. Upregulation of TIMP-1 has been described to occur concomitantly with expression of MMP-9 [23] and not unexpectedly, we found significant correlations between MMP-9 and TIMP-1 in PBMCs. We also found correlations between MMP-9 and TIMP-2 and between TIMP-1 and TIMP-2 at all levels (mRNA, protein release and plasma). An increased ratio of MMP-9/TIMP-1 has been proposed to reflect increased MMP-9 activity [24] and in a previous study, Brunner et al. [15] showed that a decreased expression of TIMP-1 in relation to MMP-9 correlated with clinical stages of CAD [15]. Our data thus suggest that TIMPs, in particular TIMP-2, are appropriately upregulated in vivo to counteract MMP-9 in stabilized post-MI patients. It is, however, known that MMP-9 is rapidly upregulated in monocytes during differentiation into macrophages [23], [25]. Therefore, we also assessed mRNA expression and protein release of MMP-9 and its inhibitors in cultured cells but found no evidence for MMP-9 dominance in post-MI patients compared with controls. Data rather indicated the opposite since patients' cells expressed significantly higher mRNA levels of TIMP-1, but not MMP-9 or TIMP-2, in vitro.

The elevated mRNA levels of MMP-9 and TIMPs in PBMCs were not reflected by protein levels in plasma. These findings are in agreement with our previous results [16] and also with a previous study by Lichtinghagen et al. [26] who compared mRNA levels of MMP-9, TIMP-1 and -2 in PBMCs with circulating protein concentrations without finding any correlations between mRNA and plasma levels.

Among blood mononuclear cells, monocytes are known to be the main producers of MMP-9 [20]. However, the expression of MMP-9 in T cells can be upregulated in immune-mediated diseases [27]. Since monocytes and T cells constitute the major infiltrating cell types in atherosclerosis, we compared the expression of MMP-9 (and TIMP-1) in monocytes and T cells by using flow cytometry. Results confirmed that monocytes was the predominant source of both MMP-9 and TIMP-1 and did not provide any evidence that these proteins were upregulated in T cells from CAD patients.

The levels of depressive symptoms were shown to be persistently elevated in post-MI patients. A few previous studies have performed longitudinal assessments of depressive symptoms up to 12 months after MI and in line with our findings, they have shown that symptoms remain elevated [28]–[30]. Furthermore, the depressive symptoms were correlated with cynical hostility. Cynical hostility is a strong and robust predictor of depressive mood [31] and has been linked to an increased risk of CAD [32], [33]. As expected, the depressive symptoms were also inversely correlated with mastery [5], [34]. Recently, the protective effect of psychological resources, such as mastery, on cardiovascular morbidity was emphasized [34].

The MMP-9 levels in plasma were not related to depressive symptoms, neither were they related to cynical hostility or mastery. This is in contrast with findings from a population-based sample of 402 individuals reporting that MMP-9 in plasma was associated with depressive symptoms (CES-D) and even more strongly with cynical hostility [5]. A proteomic investigation in a large population of psychiatric patients showed that, after insulin levels, MMP-9 levels in plasma showed the strongest association with major depression [6] and a recent study confirmed that patients with depression had persistently higher serum levels of MMP-9 compared with patients with mania or healthy controls [35]. However, plasma or serum concentrations of MMP-9 may not reflect what is going on at a cellular level [16], [26]. Previous studies by Suarez et al. [36], [37] reported that enhanced expression of cytokines and chemokines by blood mononuclear cells, suggesting a “primed” or pre-activated state of the cells, was associated with depressive symptoms in apparently healthy individuals. Interestingly, Lutgendorf et al. [7] later found that depressive symptoms (CES-D) in a population of 56 patients with ovarian cancer were related to higher MMP-9 levels in tumor-associated macrophages. In the present study, we performed extensive measurements of MMP-9 and its inhibitors in blood mononuclear cells without finding any correlations with depressive symptoms. It may be argued that depressive symptoms in our population were not severe enough. Among post-MI patients, 18% had CES-D scores≥16, a cut-off that has been associated with clinical depression [12]. This is consistent with several previous studies reporting that 15 to 20 % of post-MI patients meet the criteria for clinical depression [8]. However, not only clinical depression, but also elevated depressive symptoms may be associated with inflammation and a dose-response relationship between depressive symptoms and MMP-9 (or other inflammatory markers) has been proposed [9], [38].

One major limitation of our study is the small sample size not allowing us to evaluate the impact of clinical depression or other potential confounders like diabetes, obesity and health behaviors. Since we included only patients who were stabilized after a non-STEMI, the results may not apply to patients with a history of STEMI, neither to patients with a poor short-term prognosis after MI. On the other hand, the repeated assessments of CES-D is a strength since depressive symptoms may change over time. Also, the extensive analysis of MMP-9 and TIMPs at several levels can be regarded as a strength.

Lately, the theory that inflammation is a link between depressive symptoms and CAD has been questioned. In a retrospective case-control study, the association between depressive mood and CAD remained stable after adjustment for CRP and IL-6 levels [39]. Similarly, large prospective population studies show that associations between depressive symptoms (CES-D) or negative affect and future CAD events remain unchanged after adjustment for inflammatory markers [40], [41]. Importantly, other studies demonstrate that the association between depressive symptoms and inflammatory markers no longer remain significant after adjustment for behavioral factors, like physical inactivity, smoking and high body mass index [42], [43].

To summarize, post-MI patients exhibited overexpression of MMP-9 and TIMP mRNA in their blood mononuclear cells and also, sustained elevated depressive symptoms. Both are factors with potential impact on cardiovascular prognosis but based on our findings, they may represent two unrelated phenomena after MI.
